# A time-resolved high-throughput screening of fission yeast deletion mutants for oxidative stress resistance

**DOI:** 10.15698/mic2026.07.884

**Published:** 2026-07-22

**Authors:** Mohammadtaha Pirsalehi, Rowshan Ara Islam, Kristal Ng, Olga Xintarakou, Peter Harold Thorpe, Charalampos Rallis

**Affiliations:** Research Centre for Molecular Cell Biology, Research Centre for Evolutionary and Functional Genomics, School of Biological and Behavioural Sciences, Queen Mary University of London, Mile End Road, London, E1 4NS, United Kingdom

**Keywords:** *Schizosaccharomyces pombe*, hydrogen peroxide, genome-wide screening, growth regulation, growth/stress decoupling, *rad24*Δ, *cdt2Δ*, *lys9*Δ, oxidation, cell growth

## Abstract

Cells typically balance growth with stress responses - growing rapidly in low stress conditions and halting growth to defend against stress or to repair stress-induced damage. While numerous genome-wide screens have identified mutants resistant to oxidative stress, these have largely relied on static, end-point measurements. Here, we take a dynamic, time-resolved approach to uncover how fission yeast, *Schizosaccharomyces pombe,* adapts to oxidative stress over time. We have tracked the growth of 3,420 deletion mutants across nine time points spanning four days on both nutrient-rich solid media and media containing oxidative stress induced by hydrogen peroxide. This kinetic strategy revealed not just resistant or sensitive mutants. It allowed clustering of growth patterns across time and uncovered mutants that are capable of transiently uncoupling growth from stress response. Hydrogen peroxide induced a dose-dependent delay in colony expansion in most deletion strains, yet 15 mutants consistently maintained robust growth. These belong to different functional categories, highlighting diverse potential mechanisms ranging from altered DNA damage checkpoints to metabolic rewiring and growth regulation. By capturing dynamic trajectories rather than static outcomes, this study exposes hidden layers of growth under oxidative stress and identifies new genetic determinants of cellular resilience in fission yeast.

## INTRODUCTION

Reactive oxygen species (ROS) are inevitable byproducts of aerobic metabolism and can also arise from environmental stressors such as UV light, ionizing radiation, and hydrogen peroxide (H ${}_{2}$ O ${}_{2}$ ) 1, 2. When present in excess, ROS overwhelm the cellular antioxidant capacity, causing oxidative stress that damages DNA, proteins, and lipids. This oxidative damage contributes to ageing and underlies many pathologies, including cancer and neurodegenerative diseases 3, 4. In biotechnological contexts, oxidative stress also impairs yeast productivity by reducing viability, growth rate, and metabolic efficiency 5, 6. Yet, ROS are not merely toxic byproducts: at physiological levels, they serve essential signalling roles in growth, metabolism, and stress adaptation. Thus, maintaining ROS homeostasis is critical for sustaining cellular function 7, 8.

The fission yeast *Schizosaccharomyces pombe* is a powerful eukaryotic model for dissecting conserved stress-response mechanisms 1, 9–12. With a compact genome organised into only three chromosomes and more than 70% of its protein-coding genes having clear metazoan orthologues, *S. pombe* provides a tractable and evolutionarily relevant system for exploring fundamental principles of cell biology 13. Its fully annotated genome and comprehensive gene-deletion libraries make it particularly suitable for genome-wide screening and systems-level analysis 14, 15.

Responses to stress may require changes in gene expression. *S. pombe* integrates nutrient and stress signals primarily through two evolutionarily conserved pathways: the Target of Rapamycin (TOR) and Stress-Activated Protein Kinase (SAPK) cascades 16–19. The TOR complex 1 (TORC1) promotes growth in response to amino acid and nutrient availability 20–23. SAPKs are activated under adverse conditions such as oxidative, nutritional, heat, osmotic and other stresses and regulate translation initiation and stress responses 24, 25. These pathways act antagonistically 2: When growth-promoting TORC1 activity is high, stress protection is suppressed, and vice versa. In fluctuating environments, cells must continuously recalibrate this balance to optimise proliferation while preserving resilience 2, 16, 17.

During environmental stress, *S. pombe* mounts two levels of protection: a Core Environmental Stress Response (CESR) and stress-specific responses. The CESR, also termed the global stress response, reprograms transcription to divert cellular resources from growth toward protection and recovery, and is activated by diverse stressors, including heat, osmotic, and oxidative stress 26. In *S. pombe*, CESR activation is largely mediated by the Sty1-Atf1 mitogen-activated protein kinase cascade. Under oxidative stress, ROS indirectly activate Sty1, which phosphorylates the transcription factor Atf1, triggering the induction of genes required for repair and detoxification 1, 27–29. In parallel, *S. pombe* employs oxidant-specific regulators such as the transcription factors Pap1 and Prr1. Pap1 mediates defence against low levels of H ${}_{2}$ O ${}_{2}$ independently of Sty1-Atf1, but under high oxidative load it becomes coupled to the pathway via Srx1, a sulfiredoxin that reactivates oxidised Tpx1 -the direct activator of Pap1 30–33. Prr1 acts independently of both Sty1 and Pap1 to control a subset of oxidative stress response genes 1, 34.

Despite extensive study of oxidative stress signalling in *S. pombe*, hydrogen peroxide resistance has never been systematically examined as a primary phenotype in a genome-wide screen. Previous reports have only identified H ${}_{2}$ O ${}_{2}$ -resistant or -sensitive mutants as secondary outcomes within broader phenotypic surveys 35, 36. Two main strategies exist for genome-wide screening in *S. pombe*: pooled barcoded mutant libraries in liquid culture and arrayed mutants on solid media 15, 37–39. However, liquid-culture approaches can produce artefacts due to competition between strains of differing growth rates 40, while most solid-media studies have relied on endpoint imaging, missing the dynamic recovery phase following stress exposure 41. Because many stress-induced transcripts return to near-baseline levels even under continued stress 2, 42, such endpoint assays can yield false positives or negatives.

To overcome these limitations, we conducted a dedicated, time-resolved genome-wide deletion screen for hydrogen peroxide resistance in *S. pombe* using arrayed mutants on solid agar. Colonies were imaged repeatedly over four days, allowing us to capture dynamic growth trajectories rather than static outcomes. Beyond identifying resistant strains, our aim was to discover mutants that could uncouple growth from stress response: cells that sustain or even accelerate growth under oxidative challenge.

This question is biologically and biotechnologically important. In general, cells that grow slowly exhibit longer lifespans and greater stress tolerance 43–45, reflecting the antagonism between TOR-driven growth and stress resistance 46–48. To test whether oxidative stress-resistant mutants follow this paradigm, we performed chronological lifespan (CLS) assays, which measure the survival of non-dividing cell populations in stationary phase 49.

Identifying and characterising such resistant mutants has dual value: it provides new insight into the genetic architecture of oxidative stress defence and reveals strains with potential industrial applications. Because stress-tolerant yeasts can thrive under harsh production conditions, they represent promising platforms for biomanufacturing of biofuels, pharmaceuticals, and therapeutic proteins such as monoclonal antibodies 50–52.

## RESULTS

### Genome-wide screening of *S. pombe* deletion mutants exposed to H ${}_{2}$ O ${}_{2}$

To systematically identify the genetic determinants that alter resistance to oxidative stress, we performed a genome-wide screen of *Schizosaccharomyces pombe* deletion mutants 53. A total of 3,420 non-essential gene deletion strains were arrayed on YES agar and challenged with hydrogen peroxide (H ${}_{2}$ O ${}_{2}$ ). We initially evaluated a range of H ${}_{2}$ O ${}_{2}$ concentrations (2–8 mM). However, these doses resulted in minimal growth inhibition, likely due to cooperative stress tolerance within high-density colony arrays, where communal factors can enhance oxidative stress resistance. Based on preliminary titration experiments, 10 mM and 12 mM H ${}_{2}$ O ${}_{2}$ were selected as optimal concentrations that induced pronounced growth delay in most mutants, while still allowing differential growth among resistant strains.

**Figure 1:**
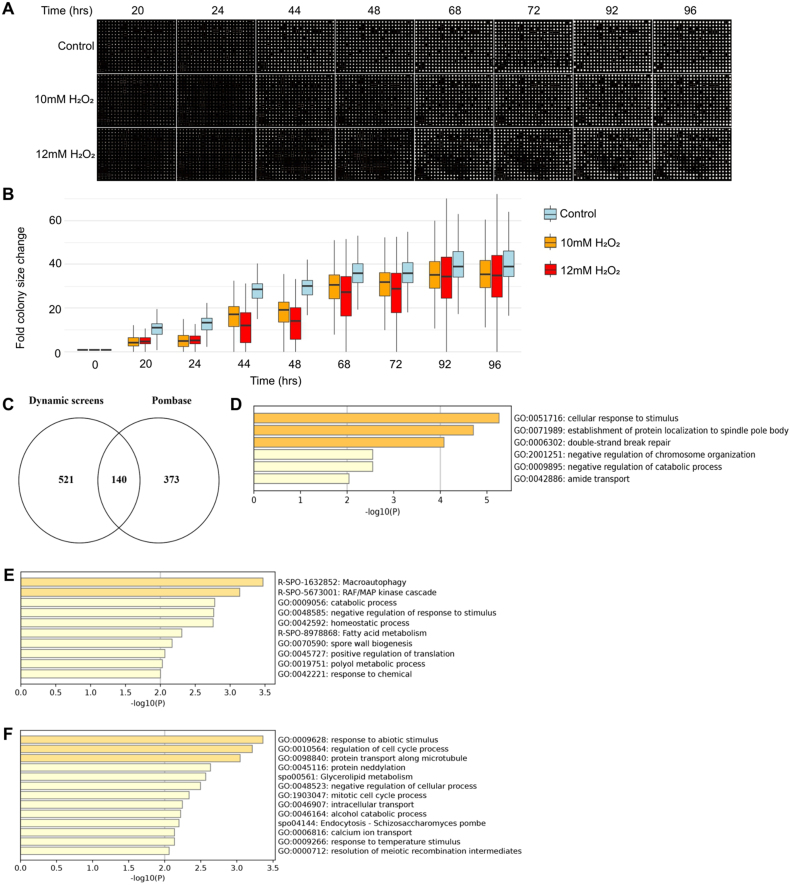
High-throughput dynamic colony growth profiling identifies oxidative stress–responsive deletion mutants. **(A)** Time-course images of colony growth for the deletion mutant library under control, 10 mM H ${}_{2}$ O ${}_{2}$ , and 12 mM H ${}_{2}$ O ${}_{2}$ . Plates were monitored across the indicated time points (20–96 h). Oxidative stress treatment resulted in delayed growth and reduced colony size compared to untreated controls, with the strongest effect at 12 mM H ${}_{2}$ O ${}_{2}$ . **(B)** Quantification of fold change in colony size across time for all deletion strains under control, 10 mM H ${}_{2}$ O ${}_{2}$ , and 12 mM H ${}_{2}$ O ${}_{2}$ conditions. Boxplots represent the distribution of colony growth trajectories at each time point, revealing a progressive divergence in growth dynamics under oxidative stress relative to controls. **(C)** Venn diagram comparing oxidative stress-responsive candidates identified in this dynamic screen with annotated oxidative stress-associated genes in Pombase. A subset of 140 genes overlapped between datasets, indicating that the time-resolved colony growth approach captures both known and previously uncharacterized oxidative stress modulators. **(D–F)** Gene Ontology (GO) biological process enrichment analysis for strains exhibiting condition-specific growth behaviors. **(D)** GO terms enriched among mutants demonstrating enhanced growth under oxidative stress relative to control. Enriched functions include cellular response to stimulus, protein localization to the spindle pole body, and DNA repair pathways. **(E)** GO terms enriched in mutants showing altered growth dynamics in the control condition, including macroautophagy, MAPK cascade signaling, metabolic regulation, and translation-associated processes. **(F)** GO terms enriched among mutants with peroxide-sensitive growth profiles, including cell cycle regulation, intracellular transport, endocytosis, calcium ion transport, and meiotic recombination resolution. Together, these analyses highlight functional modules associated with oxidative stress adaptation and growth control.

The complete deletion collection was arrayed at a density of 384 colonies per plate across nine plates per condition. Plates were imaged at nine timepoints over a 96-hour period (0, 20, 24, 44, 48, 68, 72, 92, and 96 hours), and colony growth was quantified using Gitter, an established R-based image analysis pipeline for high-throughput colony phenotyping 54. Visual inspection confirmed marked growth impairment under both oxidative stress conditions (Figure 1**A**, Fig.S1). To enable robust comparison across timepoints and strains, colony areas were normalized to initial (0 h) colony sizes, yielding growth trajectories across the time course (data available in Supplemental Tables 1 and 2). Median growth of H ${}_{2}$ O ${}_{2}$ -exposed colonies diverged from untreated controls as early as 20 h and remained significantly attenuated thereafter (Figure 1**B**), validating that 10 mM and 12 mM H ${}_{2}$ O ${}_{2}$ elicited sustained oxidative stress.

As anticipated, most mutants exhibited impaired growth relative to untreated controls, reflected by resistant ratio values < 1. The resistance ratio is calculated by dividing each mutant’s colony size on the H ${}_{2}$ O ${}_{2}$ plate by its colony size on the control plate, showing how well it grows under stress relative to normal conditions. Thus, a resistance ratio of 1 implies that a mutant is not affected by hydrogen peroxide treatment. Notably, growth inhibition correlated with the dose of H ${}_{2}$ O ${}_{2}$ : 12 mM H ${}_{2}$ O ${}_{2}$ elicited significantly lower resistance ratios than 10 mM, as assessed by the Wilcoxon rank-sum test (p < 0.003) (Figure 1**B**). We defined oxidative-stress-resistant mutants as those with a stringent resistance ratio > 1.10 under either H ${}_{2}$ O ${}_{2}$ condition. Such an applied cutoff is considered stringent as it identifies mutants that are not adversely affected by the treatment and may even exhibit enhanced growth relative to control conditions. 661 deletion strains exhibited enhanced growth in the presence of oxidative stress (Supplemental Table 3). We compared this high-confidence resistance gene set to previously annotated oxidative-stress-resistant mutants in the *S. pombe* database, Pombase 55. While there was substantial overlap, we also identified numerous strains uniquely detected by our dynamic growth-rate-based approach as well as strains previously reported but not recovered in our screen (Figure 1**C**, Supplemental tables 3 and 4). This incomplete concordance is consistent with prior observations that phenotypic screens, even when targeting identical stress responses, often display substantial variability 56. Gene Ontology (GO) enrichment analysis revealed distinct functional signatures across shared and unique mutant sets. Shared resistant strains were enriched in categories including spindle pole body organization, double-strand break repair, negative regulation of chromosomal architecture, catabolic pathways, and amide transport (Figure 1**D**). Mutants uniquely annotated in Pombase were enriched for macroautophagy, diverse catabolic processes, and cellular homeostasis pathways (Figure 1**E**). In contrast, mutants uniquely identified in our time-resolved screen showed enrichment for abiotic stress response pathways, cell-cycle regulatory proteins, and intracellular trafficking functions (Figure 1**F**). Together, these analyses support the sensitivity and complementarity of real-time growth profiling in uncovering oxidative stress resilience mechanisms beyond previously catalogued phenotypes.

In summary, our results expand the oxidative stress resistome of *S. pombe*, providing dynamic phenotypic data and revealing novel genetic regulators of H ${}_{2}$ O ${}_{2}$ tolerance. These findings highlight the modular and multifaceted nature of oxidative stress resilience and establish a resource for probing conserved stress-response pathways relevant to lifespan regulation and cellular homeostasis.

### Mutants that uncouple growth from oxidative stress response

**Figure 2:**
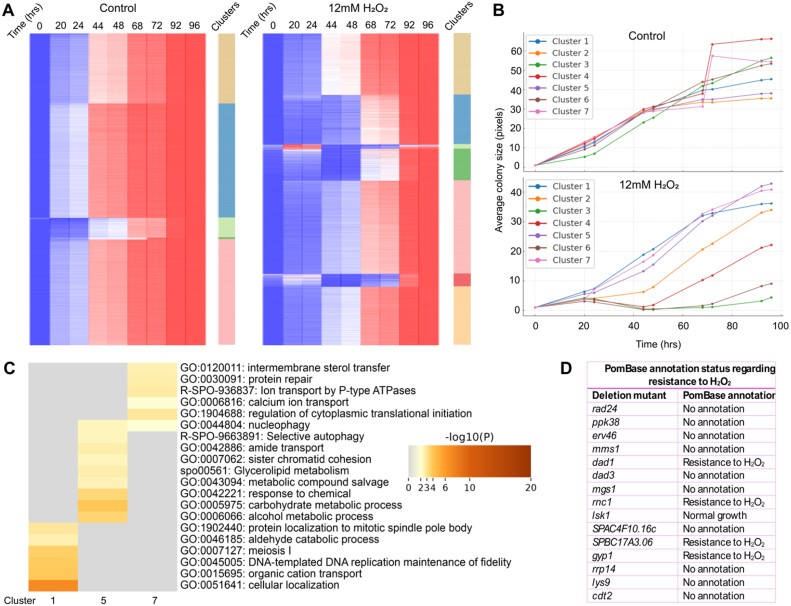
Dynamic clustering identifies oxidative stress-specific growth behaviours and mutants with enhanced stress tolerance. **(A)** Heatmap of colony growth trajectories for all deletion strains under control and 12 mM H ${}_{2}$ O ${}_{2}$ . Colours indicate fold-change in colony size (0-96 h). Seven growth clusters were identified per condition, with greater phenotypic diversity emerging under oxidative stress. **(B)** Mean growth curves of the seven clusters. Control conditions (top graph) show largely uniform growth, whereas 12 mM H ${}_{2}$ O ${}_{2}$ (bottom graph) yields distinct and variable trajectories. Clusters 1, 5, and 7 exhibit accelerated growth under stress. **(C)** GO enrichment for fast-growing stress clusters (1, 5, 7). Enriched pathways include membrane transport, protein repair, autophagy, translation regulation, and metabolism, indicating that disruption of these functions may enhance growth under oxidative challenge. **(D)** Annotation status of 15 candidate mutants selected based on stress-enhanced growth. Only four were previously noted as H ${}_{2}$ O ${}_{2}$ -resistant in Pombase, highlighting discovery of uncharacterized stress-responsive genes.

Using longitudinal colony size measurements, we performed k-means clustering to classify deletion mutants based on their growth dynamics under control and oxidative stress conditions (12 mM H ${}_{2}$ O ${}_{2}$ ). K-means clustering was conducted in the Morpheus 57, software package. This analysis yielded seven distinct groups (Figure 2**A**) showing marked heterogeneity in growth responses, with several clusters exhibiting highly variable and noisy trajectories. By contrast, mutants in control media displayed largely uniform growth (Figure 2**B**).

**Figure 3:**
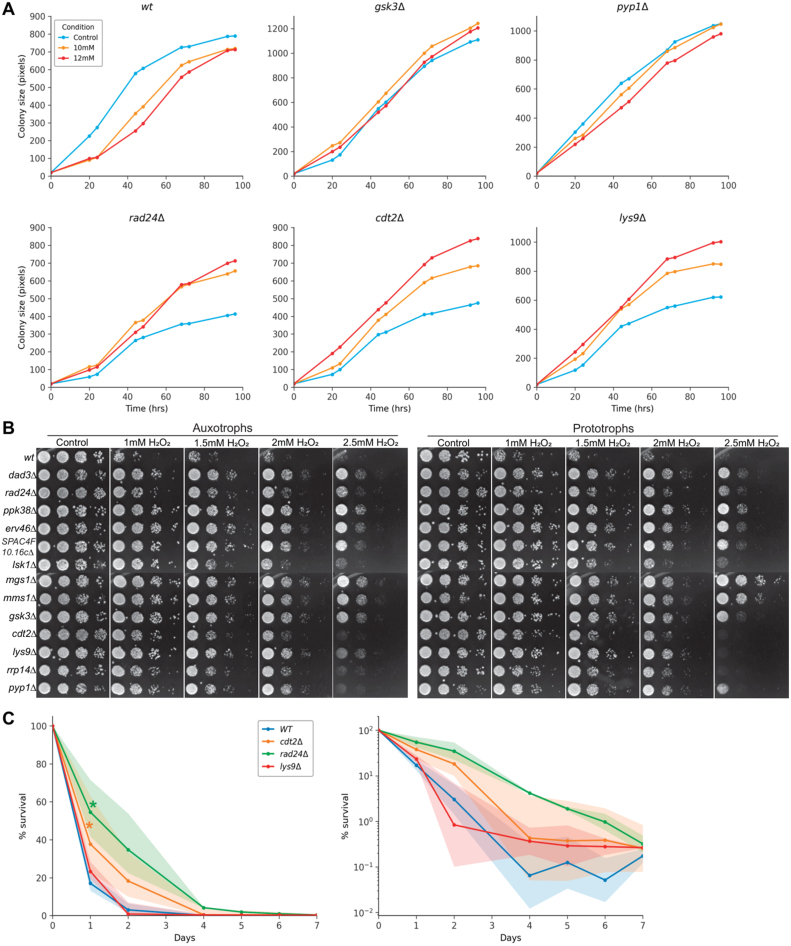
**(A)** Individual growth time-courses for *wt*, *gsk3* Δ , *pyp1* Δ , *rad24* Δ , *cdt2* Δ , and *lys9* Δ under control, 10 mM, and 12 mM H ${}_{2}$ O ${}_{2}$ . All three mutants show enhanced proliferation compared to wild type under stress. Resistant strains *gsk3* Δ and *pyp1* Δ demonstrate tolerance to hydrogen peroxide with their growth not as severely affected compared to wild type. **(B)** Spot assays validating oxidative resistance for the eleven identified non-annotated mutants. Both auxotrophic and prototrophic strains have been tested. All mutants display increased tolerance relative to wild type; *pyp1* Δ and *gsk3* Δ serve as positive controls. **(C)** Chronological lifespan analysis. *rad24* Δ and *cdt2* Δ , but not *lys9* Δ , exhibit modest lifespan extension relative to wild type. Stress tolerance and survival are not uniformly coupled. Asterisks p < 0.01, log-rank test between *rad24* Δ and *cdt2* Δ median lifespans compared to *wt.*

We annotated each cluster according to its mean growth-curve slope, categorizing them as “slow”, “normal”, or “fast” growers. Clusters 1, 5, and 7 represented the fastest-growing groups (Figure 2**B**). Gene Ontology (GO) enrichment analysis 58 revealed distinct biological themes: cluster 1 was enriched for genes involved in cellular localization, which refers to the processes that transport a molecule, protein complex, or organelle to a defined site within the cell; cluster 5 for carbohydrate metabolism, and cluster 7 for regulation of cytoplasmic translational initiation (Figure 2**C**). These results suggest that loss of genes governing specific metabolic, trafficking, or translational processes may confer a proliferative advantage under oxidative stress. To identify stress-adaptive mutants, we focused on strains whose growth was accelerated in H ${}_{2}$ O ${}_{2}$ (Figure 2**B**). Fifteen mutants met these criteria (Figure 2**D**), yet only four had been previously annotated as H ${}_{2}$ O ${}_{2}$ -resistant. Three of these strains without prior oxidative stress annotations and diverse functions, one related to DNA damage -*rad24* Δ , one to lysine metabolism *-lys9* Δ , and one to cell cycle -*cdt2* Δ , were followed up for further validations. Colony growth curves confirmed enhanced growth under peroxide, supporting the predictive value of our clustering strategy (Figure 3**A**). As controls, and for comparison we also plotted the growth of the wild-type strain as well as positive controls (*pyp1* Δ 59 and *gsk3* Δ 36) (Figure 3**A**). To independently validate oxidative stress resistance, we conducted serial dilution spot assays with fast-growing cultures at OD ${}_{600}$ =0.5 for all eleven identified strains without prior oxidative stress annotations in both original auxotrophic background as well as in prototroph state (following crosses with the wild type and selections 60). Unlike the genome-wide screen, which required higher peroxide concentrations due to mass-protection effects on solid media, validation assays used lower concentrations, enabled by pre-growth in liquid culture. This approach allowed the use of peroxide concentrations commonly employed in the field 50. Positive controls (*pyp1* Δ 59 and *gsk3* Δ 36) were included here too. All eleven candidate mutants exhibited increased tolerance compared to wild-type (Figure 3**B**).

There are links between ageing and oxidative stress 61. We have previously shown that, in contrast to slow growth, resistance to oxidative stress is not necessarily linked to long chronological lifespan in fission yeast 62. We, therefore, wondered whether our fast-growing (within stress conditions) mutants exhibit altered lifespan. Colony Forming Units (CFUs) were quantified across seven days in stationary phase. Wild-type survival dropped below 1% by day 3, and *lys9* Δ showed a similar decline. In contrast, *rad24* Δ maintained survival above 1% until day 6 (*cdt2* Δ until around day 4), indicating a modest maximal lifespan extension. In addition, *rad24* Δ and *cdt2* Δ exhibit statistically significant (^⁎^: p < 0.01, Figure 3**C**) median lifespan increase. Thus, while oxidative stress resistance is not universally associated with longevity, loss of *rad24* or *cdt2* links DNA-damage and cell-cycle regulatory pathways to improved stress tolerance and extended lifespan.

Together, these results identify a subset of deletion mutants with enhanced oxidative stress tolerance without a growth rate compromise. Our screen prioritized mutants that maintained or accelerated proliferation under peroxide challenge.

## DISCUSSION

Our genome-wide screen identified 15 candidate mutants resistant to hydrogen-peroxide stress and able to grow fast enriching the list of already known mutants with such behaviour. GO enrichment of faster-growing clusters under 12 mM H ${}_{2}$ O ${}_{2}$ highlighted deletions in genes involved in cellular localization, carbohydrate metabolic processes, and regulation of cytoplasmic translational initiation, suggesting that loss of these functions can facilitate faster growth under oxidative stress. Long lifespan was observed for the resistant *rad24* Δ and *cdt2* Δ strains whereas *lys9* Δ did not, indicating that oxidative stress tolerance does not necessarily correlate with altered lifespan.

Our observations align with the core environmental stress response modules (CESR) in yeasts. Stress exposure induces a stress response while repressing ribosome biogenesis and other growth programs 2, a shift that depends on dose and time and is largely regulated by the Sty1-Atf1 MAPK pathway 26 in *S. pombe* (as well as Pap1 during oxidative stress 1). Even under persistent stress, cells adapt to stress, and growth eventually resumes, explaining the recovery phase we observed between 48–68 h on plates (ratios trending back toward 1) 1, 2. As confirmed by our validation assay, *rad24* Δ *, lys9* Δ *, and cdt2* Δ are resistant to H ${}_{2}$ O ${}_{2}$ . The “enhanced growth” seen on plates likely reflect colony-level uncoupling of growth from stress response in these conditions. Dense communities can detoxify H ${}_{2}$ O ${}_{2}$ and lower the effective dose for interior cells 63. Yeast colonies are known to behave differently in communities. This difference in H ${}_{2}$ O ${}_{2}$ exposure between agar and liquid cultures explains why colonies can appear to outgrow stress while liquid cultures do not 64, 65.

GO analysis points to three biological processes whose deletion could improve growth under H ${}_{2}$ O ${}_{2}$ . First, deletions that shift carbohydrate metabolism away from glycolysis can divert carbon into the oxidative pentose-phosphate pathway (PPP), boosting NADPH production to fuel glutathione and peroxiredoxin systems; key glycolytic steps or oxidant-mediated inhibition of glycolytic enzymes GAPDH/PK, shift towards PPP, and improve oxidant resistance 66. Second, under oxidative stress, cells suppress global translation and counteract oxidative stress *via* translational control of a subset of mRNAs 67. Thus, deleting positive regulators of global protein translation initiation can result in a growth advantage when cells are exposed to hydrogen peroxide 68, 69. Finally, it is known that Sty1-Atf1 localization is critical upon stress, the MAPK Sty1 accumulates in the nucleus and cooperates with Atf1p to initiate stress-gene transcription 70. Therefore, it may plausible that deleting genes facilitating the cellular localization of Sty1 upon stress may result in faster-growing cells; if so, the usual downregulation of TORC1 may fail to occur, consistent with reports that stress inhibits TORC1 in fission yeast 29, 68, 69.

We validated that *rad24* Δ *, lys9* Δ , and *cdt2* Δ are resistant to H ${}_{2}$ O ${}_{2}$ . *lys9* encodes saccharopine dehydrogenase, an enzyme involved in lysine biosynthesis; loss of *lys9* causes lysine auxotrophy 71. Importantly, the rich YES medium includes lysine as a supplement (225 mg/l), so *lys9* Δ cells have access to external lysine. In budding yeast, adding extracellular L-lysine triggers “lysine harvesting”; Cells import far more lysine than they need for growth, which allows them to save NADPH and boost glutathione (GSH) 72. This re-routing increases cellular GSH and improves resistance to oxidants. We hypothesized that *lys9* Δ may result in cells importing lysine from YES, saving NADPH for antioxidant systems and enhancing H ${}_{2}$ O ${}_{2}$ tolerance 72, 73. This led us to wonder whether this behaviour is related to lysine metabolism-related genes or is unique to *lys9* mutants. Nine genes beyond *lys9* are reported (pombase) to be implicated in lysine metabolism including *idh1*, *idh2*, *lys1*, *lys12*, *lys2*, *lys3*, *lys4*, *lys7* and *msk1.* Nevertheless, none of these show the same growth pattern indicating that is related to Lys9 function as a saccharopine dehydrogenase with NADP+ and L-glutamate-forming activity.

*rad24*, a 14-3-3 protein, binds the mitotic activator Cdc25 and helps the G2/M checkpoint; when Cdc25 is phosphorylated by checkpoint/stress kinases, *rad24* keeps it away from the nucleus to delay mitosis 74, 75. Based on this checkpoint logic, we hypothesize that *rad24* Δ mutants may be failing to arrest in G2 during oxidative stress, rather than improving H ${}_{2}$ O ${}_{2}$ detoxification, they keep dividing despite DNA damage.

Cdt2 is the substrate receptor of the CRL4-Cdt2 E3 ubiquitin ligase 76, 77. In *S. pombe*, CRL4-Cdt2 is recruited when Proliferating Cell Nuclear Antigen (PCNA) is loaded on DNA during S-phase or repair and targets specific proteins for ubiquitylation and degradation. By removing these targets, CRL4-Cdt2 prevents re-replication and helps maintain adequate dNTP pools. Loss of *cdt2* leaves these proteins in place, extends S-phase, activates checkpoints, and often slows proliferation 76, 77. In our liquid assay, *cdt2* Δ showed slow growth raising the possibility that cells have spent longer in S-phase. This raised the possibility that S-phase extension could cause the observed H ${}_{2}$ O ${}_{2}$ resistance, consistent with the general trend that a slow growth rate contributes to oxidative stress tolerance 45. Nevertheless, further examination of growth patterns of deletion mutants with increased S-phase duration such as those for *dfp1*, *noc3*, *pli1*, *trx1* did not support this hypothesis with the exception of *nup132*, coding for a nucleoporin 78.

In our CLS assays, *rad24* Δ (and less extensively *cdt2* Δ ) lived longer, while *lys9* Δ lifespan was similar to the wild type. This is opposite to Pombase annotation, which lists *cdt2* Δ 79, *rad24* Δ 80 as short-lived and *lys9* Δ 80 as long-lived. Those data come mainly from pooled, competitive screens and flow-cytometry–based viability measurements; pooled designs can be affected by outgrowth effects, whereas our non-competitive CFU assay avoids this issue and may explain the difference 80, 81. Mechanistically, there is no evidence in the literature that *cdt2* is involved in TORC1 regulation. In addition, *rad24* promotes Wee1 stability in fission yeast, and TORC1 inhibition reduces Wee1 levels; thus, a *rad24* deletion could further destabilize Wee1, but a fall in Wee1 has not been shown to inhibit TORC1 in *S. pombe* 82. Thus, we cannot yet link the longer CLS of *cdt2* Δ or *rad24* Δ to TORC1 inhibition 46, 62. A plethora of compounds and other pathways have been shown to increase chronological lifespan of fission yeast including the Pmk1, PKA and Sty1, Ecl family genes pathway, Php complex and Clg1-Pef1 pathway 83, 84. As in the case of the TORC1 pathway, we could not draw a direct connection of the identified mutants with these pathways. Finally, although *cdt2* Δ and *rad24* Δ combine H ${}_{2}$ O ${}_{2}$ resistance with longer CLS in our study, *lys9* Δ does not, indicating that stress resistance and longevity often coincide, but not always.

Altogether, we have identified a large number of mutants (521 novel ones, Figure 1**C**, Supplemental Tables 3 and 4) exhibiting resistance to hydrogen peroxide using our dynamic screen. As mentioned, incomplete overlap with mutants already reported within Pombase as resistant to hydrogen peroxide (373 mutants) agrees with prior observations: phenotypic screens, even when targeting identical stress responses, can display substantial variability 56. The parameters of our screen differ from previous ones in terms of the concentration of the stressor used (prior concentrations used were 0.5 and 1 mM) 39. In addition, we found 15 mutants that grow well (or even better) in oxidative stress conditions, thus, decoupling growth from stress gene expression programmes. Although the exact mechanisms behind this resistance are not within the scope of this manuscript, this study enhances our knowledge base of resistant mutants. Expanding our efforts beyond hydrogen peroxide, we are now able to engineer fission yeast to resist different types of stress and utilise them towards biotechnological applications 50, 52. Our results show that following growth over time can help uncover meaningful phenotypes that may be missed in single-time-point screens. While a single late timepoint recovers most of the resistant mutants (457), time-resolved analysis was essential to identify a substantial fraction ( ∼ 30%) of resistance phenotypes (a total of 661). The results also suggest that oxidative stress resistance is not always linked to growth rates and CLS and that different pathways may support survival in distinct ways. This work provides a practical method for identifying oxidative stress-related genes and offers a clear starting point for deeper investigation into the cellular mechanisms that help fission yeast manage environmental stress.

## MATERIALS AND METHODS

### Media preparation

Yeast was grown in YES medium (3% glucose and 225mg/l for each of Adenine, Uracil, Leucine, Histidine and Lysine) throughout. To prevent contamination during the 4-day-long imaging course G418 was added. Three conditions were used throughout: control, 10 mM H ${}_{2}$ O ${}_{2}$ , and 12 mM H ${}_{2}$ O ${}_{2}$ . Hydrogen peroxide (30% w/v; Sigma-Aldrich) was added to molten medium to achieve the desired concentrations.

### Library preparation and phenotypic screening

50 mL media have been used for each Singer RoToR plus plate. A copy of the *S. pombe* Bioneer deletion library (version 5; 3,420 mutants) was arrayed in 384-format on YES plates using a RoToR robot (Singer Instruments) and incubated for 48 h at 32 ${}^{\circ }$ C. These served as templates for pinning onto control and H ${}_{2}$ O ${}_{2}$ -containing plates. Pinning was performed at 3% pressure with short 384-pins and no source mixing to minimize biomass, as dense colonies show increased stress tolerance 65. Plates were incubated at 32 ${}^{\circ }$ C for and phenotypes were monitored for 4 days. Colony growth was imaged at 0, 20, 24, 44, 48, 68, 72, 92, and 96 h using an Epson Perfection V850 Pro scanner (400 dpi, reflective mode). Images were pre-processed for analysis with the R package Gitter 54 quantifying colony areas. Quantification data were linked to fission yeast gene IDs based on the plate geography.

### Data analysis

Colony size data were normalized to 0 h (growth fold-change) and to the corresponding control (resistance ratio). Data visualization and statistical tests were performed in RStudio using *ggplot2* 85. Resistance ratios between 10 mM and 12 mM H ${}_{2}$ O ${}_{2}$ were compared using the Wilcoxon rank-sum test. Heatmaps of growth patterns were generated in Morpheus 49, and Gene Ontology (GO) enrichment of fast-growing mutants was assessed using Metascape 58. Fifteen mutants exhibited resistance ratios > 1.15 under both peroxide concentrations at all time points. Using *k*-means clustering (Morpheus), mutants were grouped into seven clusters, and average growth fold-change across time was plotted. Mutants that grew slowly in control but rapidly under 12 mM H ${}_{2}$ O ${}_{2}$ were prioritized for validation.

### Validation of resistance to hydrogen peroxide by spot assay

Candidate mutants and wild-type strains were grown in YES broth to mid-log phase (OD ${}_{600}$ = 0.5), incubated for 30 min, and serially diluted 10-fold. Dilutions were spotted on YES plates containing H ${}_{2}$ O ${}_{2}$ and incubated at 32 ${}^{\circ }$ C for 48 h. Growth was imaged using a Vilber Lourmat FUSION Solo S system. *pyp1* Δ 53 and *gsk3* Δ 30 strains served as positive controls.

### Chronological lifespan assay

Mutants were cultured in YES broth at 32 ${}^{\circ }$ C until stationary phase. Daily serial dilutions were plated on YES agar to determine colony-forming units (CFUs). Day 0 CFUs were defined as 100% survival, and subsequent counts were expressed relative to this value. CLS curves were generated from three biological replicates, with at least three technical replicates per time point.

## SUPPLEMENTAL MATERIAL

All supplemental data for this article are available online at http://www.microbialcell.com/researcharticles/2026a-pirsalehi-microbial-cell/.

Click here for supplemental data file.

Click here for supplemental data file.

Click here for supplemental data file.

Click here for supplemental data file.

## CONFLICT OF INTEREST

The authors declare that they have no competing interests.

## ABBREVIATIONS

CESR – core environmental stress response

CFUs – colony forming units

CLS – chronological lifespan

ROS – reactive oxygen species

SAPK – stress-activated protein kinase

TOR – target of rapamycin

TORC – TOR complex
