# Supplementary material for: A time-resolved high-throughput screening of fission yeast deletion mutants for oxidative stress resistance
Source: Microb Cell. 2026 Jul 22;13:304–13. doi: 10.15698/mic2026.07.884 (PMC13392485; doi:10.15698/mic2026.07.884)
Supplement: Supplementary file 1 [file mic-13-304-s01.pdf]

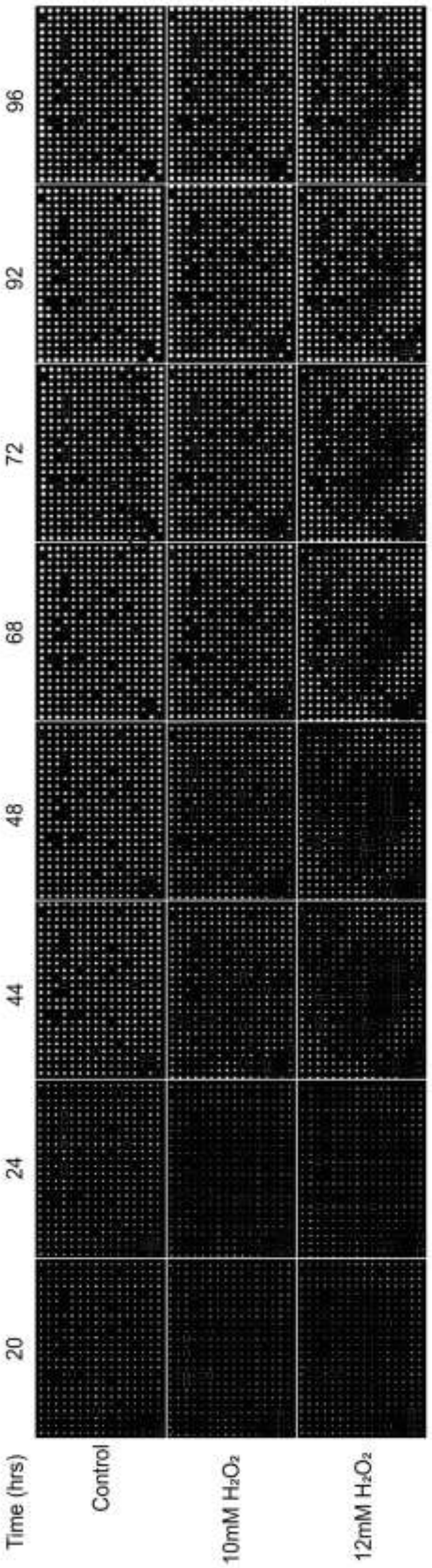

Supplemental Figure S1: Enlarged version of Figure 1A panel towards better evaluation of growth defects on plates. Note that the panel shows only one plate as a representative example and does not recapitulate the results of the screens.

Supplemental Table 1. List of all strains and their resistance ratios across all timepoints examined in 10 mM hydrogen peroxide.

Supplemental Table 2. List of all strains and their resistance ratios across all timepoints examined in 12 mM hydrogen peroxide.

Supplemental Table 3. List of the 661 strains with a resistance ratio above 1.1 in one or more of the timepoints examined.

Supplemental Table 4. Lists of the genes reported as resistant to hydrogen peroxide through the dynamic screen only (541 genes), genes reported in pombase only (373 genes) and common ones (140 genes).
